# The Role of Temporal Contingency and Integrity of Visual Inputs in the Sense of Agency: A Psychophysical Study

**DOI:** 10.3389/fpsyg.2021.635202

**Published:** 2021-04-01

**Authors:** Hiroaki Mizuhara, Peter Uhlhaas

**Affiliations:** ^1^Institute of Neuroscience and Psychology, University of Glasgow, Glasgow, United Kingdom; ^2^Graduate School of Informatics, Kyoto University, Kyoto, Japan; ^3^Department of Child and Adolescent Psychiatry, Charité Universitätsmedizin, Berlin, Germany

**Keywords:** sense of agency, comparator model, consciousness, visual integrity, temporal contingency, mutual information

## Abstract

The sense of agency is a subjective feeling that one's own actions drive action outcomes. Previous studies have focused primarily on the temporal contingency between actions and sensory inputs as a possible mechanism for the sense of agency. However, the contribution of the integrity of visual inputs has not been systematically addressed. In the current study, we developed a psychophysical task to examine the role of visual inputs as well as temporal contingencies toward the sense of agency. Specifically, participants were required to track a target on a sinusoidal curve on a computer screen. Visual integrity of sensory inputs was manipulated by gradually occluding a computer cursor, and participants were asked to report the sense of agency on a nine-point Likert scale. Temporal contingency was manipulated by varying the delay between finger movements on a touchpad and cursor movements. The results showed that the sense of agency was influenced by both visual integrity and temporal contingency. These results are discussed in the context of current models that have proposed that the sense of agency emerges from the comparison of visual inputs with motor commands.

## Introduction

The sense of agency is the subjective feeling that one's actions drive behavioral outcomes and it is crucial for discriminating between self and non-self. Integration of motor predictions with visual inputs of action outcomes is a potential mechanism to induce the sense of agency (Haggard, 2005, 2017; David et al., 2008; Wen, 2019).

Several studies have focused on the contribution of temporal contingencies between action and visual inputs. For example, Farrer et al. (2008a) examined the sense of agency during a task in which short delays between a joystick manipulation and a virtual arm movement were embedded (Farrer et al., 2008a). The results showed that increased delays reduced the sense of agency, a finding which has been replicated in several studies (Sirigu et al., 1999; Tsakiris et al., 2005; Haggard, 2017).

Implicit judgments have also been used to investigate the sense of agency in intentional binding tasks (Haggard et al., 2002). Specifically, intentional binding tasks examine the effects of a voluntary action on the time intervals by comparing to a baseline, which was, for example, an involuntary action induced by transcranial magnetic stimulation, or irrelevant passive sounds, suggesting a relationship between intentional binding and volition (Haggard et al., 2002). Previous studies have highlighted the role of temporal contingencies (Moore et al., 2009), suggesting that intentional binding is sensitive to causal relationship between action and sensory inputs. These results support the comparator model of motor control (Haggard, 2005) in which the sense of agency is generated when the difference between motor predictions and the incoming visual inputs is decreased.

In addition to temporal contingency, sensory inputs may also crucially modulate the sense of agency. In scenarios where temporal contingency is increased, visual inputs that do not match the motor command would diminish the sense of agency. This perspective is supported by a previous study in which a decrease in the sense of agency was demonstrated when ambiguity was increased by spatial perturbation during the occlusion of a virtual hand movement (Preston and Newport, 2008).

Two additional studies also addressed the role of visual inputs directly (Asai, 2015; Miyawaki and Morioka, 2020). Specifically, the authors introduced a manipulation of a computer cursor which involved a flicker at 4 Hz. The results showed that both temporal contingency as well as the integrity of visual inputs diminished the sense of agency. However, in these studies, visual integrity and temporal contingency were not investigated in the same experimental design, raising the question whether these factors interact as well as their respective contribution toward the sense of agency.

In the current study, we developed a psychophysical task to examine the contribution of temporal contingency as well as the integrity of visual inputs toward the sense of agency. Participants were asked to trace a sinusoidal curve on a computer screen by moving a cursor with a touchpad. We manipulated visual inputs by temporally occluding the cursor in the middle of the peaks and troughs of the curve. Moreover, temporal contingency was manipulated by introducing short delays between participants' finger movements and visual inputs of the cursor movement, consistent with previous studies (David et al., 2008; Farrer et al., 2008a, 2013; Kawabe et al., 2013; Wen, 2019). In addition to confidence ratings, we also used an information theoretical approach to quantitatively observe the additive effects of temporal contingency and visual integrity on the sense of agency.

## Materials and Methods

### Participants

Forty right-handed participants (24 females and 16 males; mean age: 24.2 ± 1.5 years) participated in the experiment after providing written informed consent. The sample size was determined based on previous studies (e.g., Moore et al., 2009; Asai, 2015). An institutional ethics committee approved the experiment at the University of Glasgow (protocol # 300180086).

### Apparatus

An in-house MATLAB software with Psychtoolbox-3 (http://psychtoolbox.org/) was used for stimuli presentation. The refresh rate of the display was 60 Hz and the drawing of stimuli was refreshed every 33.3 ms (30 Hz). Stimuli were presented on a laptop screen (Surface Laptop, Microsoft, USA) ~60 cm away from the participants. A touchpad on the laptop was used to detect the participant's finger's x and y coordinates on the screen. The experimental software is available from our web site (https://u.kyoto-u.jp/ltecm).

### Stimuli

**Figure 1:**
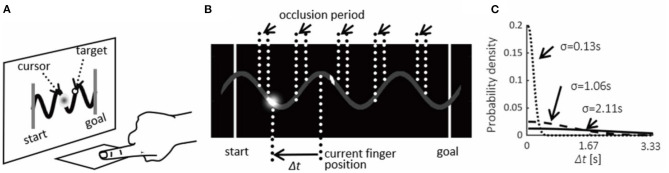
Experimental task. **(A)** Target moved on a sinusoidal curve for 3 s per trial. Participants were required to track the target by moving their finger on a touchpad to manipulate a cursor on a computer screen. The cursor movement followed participants' finger movement in half of the trials (“self condition”) while in the other trials, a recorded trajectory was presented (“non-self condition”). **(B)** Manipulation of visual integrity: the cursor always appeared at the peaks and troughs and disappeared at the middle between the peaks and troughs during 10, 40, or 70% duration of a cycle of the sinusoidal curve. **(C)** Manipulation of temporal contingency: the temporal delays (Δ*t*) were perturbed according to a probability derived from a Gaussian distribution with three different variances (σ^2^).

A target (width = 0.12°) moved from left to right over a gray sinusoidal curve (three cycles, length = 6.2°, amplitude = 0.52°, line width = 0.12°) (Figure 1A). Participants manipulated the location of a cursor by moving their right index or middle finger over a touchpad.

### Procedure

Participants were required to trace a white dot as accurately as possible by moving a cursor on a computer screen. Participants were required to indicate how confidently they felt that the cursor was moved by themselves by responding on a nine-point Likert scale. Previous studies used a 5, 7, or 9-point Likert scale (Preston and Colman, 2000). Since one of the objectives of the current study was to calculate MI-values and smaller bin numbers can lead to an underestimation of MI (Seok and Seon Kang, 2015), a 9-point Likert scale was employed.

Temporal delays were introduced between finger and cursor movements according to the absolute value of the Gaussian distributions (SD = 4, 32, or 64 frames, i.e., 0.13, 1.06, or 2.11 s) (“temporal contingency”) (Figures 1B,C). The cursor was also removed between peaks and troughs of the sinusoidal curve during 10, 40, or 70% of the duration of a cycle (“visual integrity factor”) (Figure 1B).

The movement of the cursor corresponded to participants' finger movements only in half of the trials (“self condition”). During the other half, the trajectory of the cursor corresponded to a recorded trajectory of the participant (“non-self condition”). Temporal delays for the non-self condition were introduced to the instantaneous position of the recorded finger trajectory. The temporal delay was not fixed, but varied according to the probability distribution shown in Figure 1C from moment to moment as the finger moved on a sinusoidal curve. “Self” and “non-self” conditions were randomized.

Prior to the experiment, the following instructions were given to participants: “The movement of the cursor is corresponding to your finger movement only on half of the trials. During the other half of the trials, it moves automatically, that is, independent of your finger movements. These conditions occur randomly.”

Each trial was initiated at 0.5, 1.0, 1.5, or 2.0 s following the response. The experiment consisted of three blocks of 15 min (72 trials each), and each condition randomly appeared eight times in one block. Before the experiment, the participants performed short practice blocks (16 trials, <3 min).

### Data Analysis

Sense of agency was computed by subtracting confidence ratings between the self- and non-self condition. We also analyzed confidence ratings for each experimental condition.

MI-values was computed between confidence ratings and levels of temporal contingency or visual integrity factors with the “MItoolbox” (Brown et al., 2012). Briefly, MI is a quantitative measure determining whether or not two events occurred at the same time (Phillips and Craven, 2000; Schyns et al., 2011; Seok and Seon Kang, 2015). MI analyses assessed whether ratings of self-agency varied according to the level of temporal contingency or visual integrity factors. MI values were calculated on individual ratings for each trial.

Accuracy of finger movements was defined as a distance between a finger position and the target by calculating the mean Euclidean distances between them over each cycle of the sinusoidal curve on the screen. The distance was expressed in pixels of monitor resolution, where the total length of a three-cycles sinusoidal curve (~6.2°) was 564 pixels. In this analysis, the accuracy was analyzed for each period of the sinusoidal curve shown in Figure 1B.

### Statistical Analysis

A two-way within-subject analysis of variance (ANOVA) was used to analyze behavioral data, whereby the temporal delay constituted the “temporal contingency factor” with three levels (0.13, 1.06, and 2.11 s) and the occlusion of the cursor with three levels (10, 40, and 70% occlusion ratios) the “visual integrity factor.” These two factors were the repeated measure factors, and the subjective ratings of agency and the difference in the ratings between the self and non-self conditions were the dependent variables. The violation of sphericity in repeated measures ANOVA was corrected by the Greenhouse-Geisser method to compute statistical *p*-values (*p*_*GG*_; Greenhouse and Geisser, 1959).

In the ANOVA for MI, temporal contingency as well as visual integrity and the self/non-self conditions were the two repeated measure factors, with MIs as the dependent variable.

The accuracy of the finger movements was compared between conditions using a four-way ANOVA. The factors were visual integrity (3 levels), temporal contingency (3 levels), self- and non-self conditions (2 levels), and the periods of the sinusoidal curve (3 levels).

## Results

### Sense of Agency

**Figure 2:**
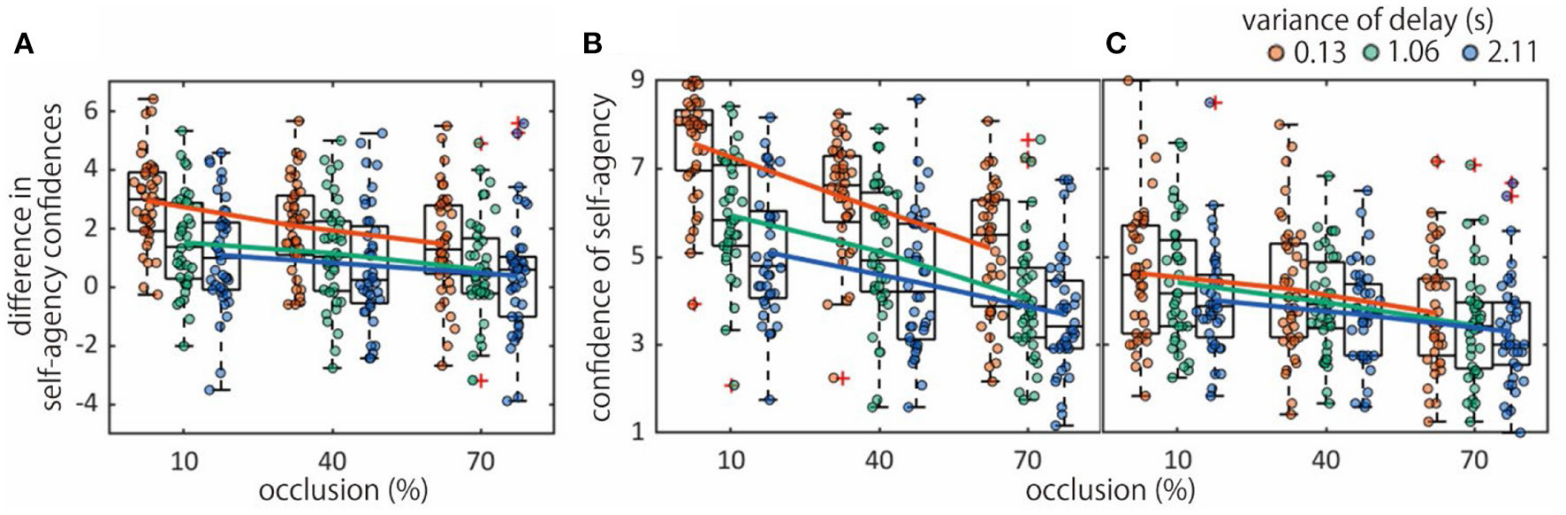
Sense of agency according to temporal contingency and visual integrity factors. **(A)** Difference in confidences of self- and non-self judgements. **(B)** Confidence ratings in the self condition. **(C)** Confidence ratings in the non-self condition.

The sense of agency was modulated by temporal contingency [*F*_(2, 78)_ = 47.0, *p*_*GG*_ < 0.0001, η_*p*_^2^ = 0.17] and visual integrity [*F*_(2, 78)_ = 22.8, *p*_*GG*_ < 0.0001, η_*p*_^2^ = 0.090] (Figure 2A). The results show that the sense of agency decreased as visual integrity and the temporal contingency were reduced. Also, there was an interaction between the factors [*F*_(4, 156)_ = 2.55, *p*_*GG*_ = 0.041, η_*p*_^2^ = 0.011], suggesting that both temporal contingency and visual integrity factors impacted on the sense of agency. Both manipulations modulated confidence ratings in both the self- and non-self conditions [for self condition, see Figure 2B; temporal contingency: *F*_(2, 78)_ = 91.0, *p*_*GG*_ < 0.0001, η_*p*_^2^ = 0.024; visual integrity: *F*_(2, 78)_ = 127.2, *p*_*GG*_ < 0.0001, η_*p*_^2^ = 0.021] [for non-self condition, see Figure 2C; temporal contingency: *F*_(2, 78)_ = 10.5, *p*_*GG*_ = 0.001, η_*p*_^2^ = 0.0032; visual integrity: *F*_(2, 78)_ = 18.0, *p*_*GG*_ < 0.0001, η_*p*_^2^ = 0.0080]. The ANOVA in the self condition was characterized by an interaction between the temporal contingency and visual integrity factors [for self condition, *F*_(4, 156)_ = 4.93, *p*_*GG*_ = 0.001, η_*p*_^2^ = 0.00097] [for non-self condition, *F*_(4, 156)_ = 0.78, *p*_*GG*_ = 0.54, η_*p*_^2^ = 0.00015].

### Mutual Information

**Figure 3:**
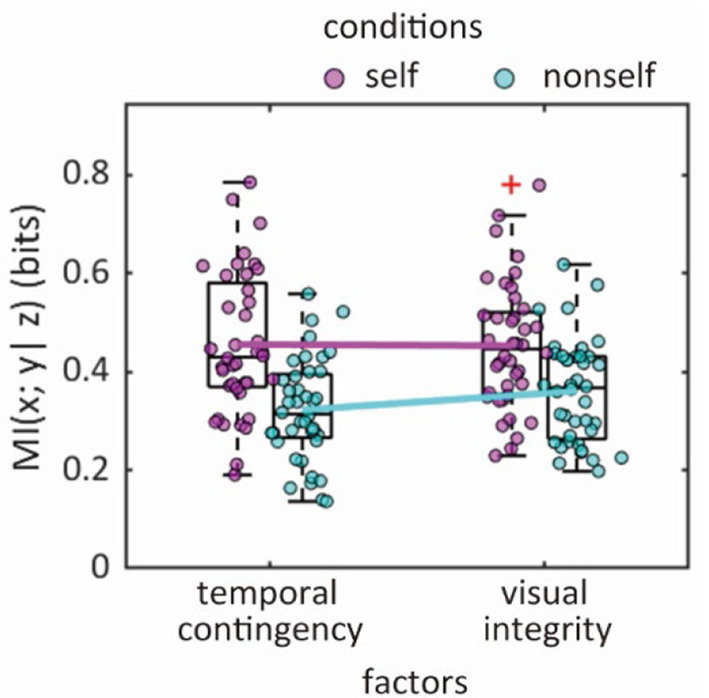
Impact of confidence of self-agency by temporal contingency and visual integrity. Mutual information (MI) values for confidence of agency in relationship to levels of temporal contingency and visual integrity. Lines represent means of MI between participants. Each plot represents MI-data for individual participants.

MI was significantly higher in the self condition (0.45 bits) than in the non-self condition (0.34 bits) [*F*_(1, 39)_ = 32.2, *p* < 0.0001, η_*p*_^2^ = 0.020], but there was no difference between the temporal contingency and visual integrity factors [*F*_(1, 39)_ = 1.38, *p* = 0.25, η_*p*_^2^ = 0.00050] (Figure 3). However, a trend toward an interaction was observed [*F*_(1, 39)_ = 3.73, *p* = 0.061, η_*p*_^2^ = 0.00066]. A *post-hoc* analysis revealed a main effect of the temporal contingency and visual integrity factors in the non-self condition (*p* = 0.022, Cohen's *d* = 0.37), while there was no difference between these factors in the self condition (*p* = 0.90, *d* = 0.020). Accordingly, in the self condition, temporal contingency and visual integrity factors equally affected the sense of agency.

### Finger Movement Accuracy

**Figure 4:**
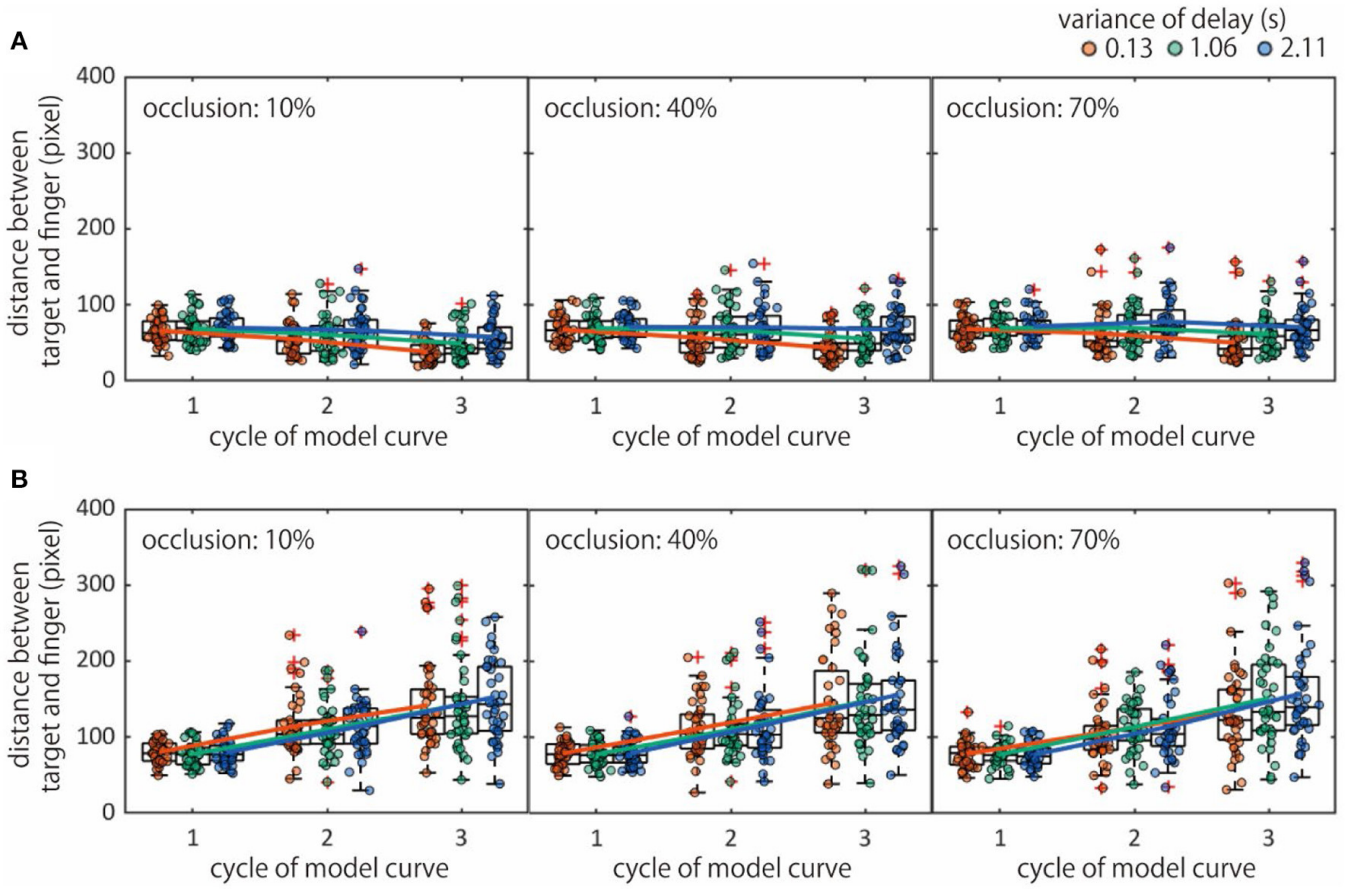
Accuracy of finger movement trajectories. Distance between finger and target in the self- **(A)** and non-self **(B)** condition.

The accuracy of finger movement trajectories was related to temporal contingency [*F*_(2, 76)_ = 21.39, *p*_*GG*_ < 0.0001, η_*p*_^2^ = 0.0020] (Figure 4). In addition, a trend for the visual integrity factor was also observed [*F*_(2, 76)_ = 2.88, *p*_*GG*_ = 0.063, η_*p*_^2^ = 0.00034] and there was an interaction with the self-/non-self conditions [*F*_(2, 76)_ = 5.41, *p*_*GG*_ = 0.007, η_*p*_^2^ = 0.00039]. We performed a *post-hoc* analysis which showed a decrease of the accuracy with a reduction of visual integrity [for self condition: *p* < 0.046, *d* > 0.34; for non-self condition: *p* > 0.860, *d* < 0.008] and temporal contingency [for self condition: *p* < 0.0001, *d* > 0.29; for non-self condition: *p* > 0.102, *d* < 0.12] only in the self condition.

## Discussion

Previous studies have focused on the relationship between temporal contingency and sense of agency (Tsakiris et al., 2005; Farrer et al., 2008a,b; Farrer et al., 2013; Khalighinejad and Haggard, 2016). In this study, we introduced a novel psychophysical task to examine whether the sense of agency can be modulated by the integrity of visual inputs as well as by the temporal contingency between visual inputs and motor responses. In line with previous studies (Farrer et al., 2008a, 2013; Khalighinejad and Haggard, 2016), our results confirmed that the sense of agency was modulated by changes in temporal contingency.

Importantly, we also found an effect of visual integrity whereby a decrease in visual integrity resulted in a decrease in the sense of agency. These data are in line with previous work by Asai (2015) as well as Miyawaki and Morioka (2020) who conducted experiments which required participants to trace a sinusoidal curve and degraded visual inputs through different flicker frequencies (4 and 8 Hz).

One potential framework for the effects of temporal contingency and visual integrity is the comparator model (Haggard, 2005, 2017), which highlights that the sense of agency occurs when a visual input coincides with a motor command. When visual integrity is reduced, less visual cues are available for evaluating the sense of agency and there is a stronger reliance on motor prediction for attributional judgments. This hypothesis is supported by the interaction between visual integrity and temporal contingency factors in the self condition. When sufficient visual cues were available, self-initiated movements could be compared with motor predictions to facilitate attributional judgments.

Further analyses using an information theoretical approach revealed that visual integrity had a similar impact as temporal contingency on the sense of agency in the self condition. The finding that the effects of these factors were identical is novel finding that was not addressed by previous studies (Asai, 2015; Miyawaki and Morioka, 2020). We also found that visual integrity had a greater impact on agency judgments than temporal contingency alone, suggesting that the sense of agency emerges from a combination of visual inputs and the temporal contingency between action and sensory information.

A limitation of the current study is that the temporal contingency factor did not have an impact on the accuracy of finger trajectories in the non-self condition. On the other hand, a clear temporal contingency effect was observed on confidence ratings in the non-self condition. One possibility is that the discrepancy between the targets and finger positions was too large, thus introducing a ceiling effect.

In conclusion, our study shows that the sense of agency is not simply determined by the temporal contingency between action and visual information, but also by the integrity of visual inputs itself. In the comparator model, discrepancy in the comparison becomes larger not only when visual inputs and actions are temporally misaligned, but also when visual inputs are degraded. The integrity of visual inputs could therefore constitute an important factor for the sense of agency, both alone and in combination with the temporal contingency of actions.

## Data Availability Statement

The datasets presented in this study can be found in online repositories. The names of the repository/repositories and accession number(s) can be found below: The Kyoto University Research Information Repository (KURENAI) (https://repository.kulib.kyoto-u.ac.jp/dspace/?locale=en).

## Ethics Statement

The studies involving human participants were reviewed and approved by Institute of Neuroscience and Psychology, University of Glasgow (protocol #300180086). The patients/participants provided their written informed consent to participate in this study.

## Author Contributions

HM and PU contributed to the conception and design of the study. HM performed the experiment and statistical analysis and wrote the first draft of the manuscript. All authors contributed to manuscript revision, read, and approved the submitted version.

## Conflict of Interest

The authors declare that the research was conducted in the absence of any commercial or financial relationships that could be construed as a potential conflict of interest.
